# Mechanistic Insights into Validoxylamine A 7'-Phosphate Synthesis by VldE Using the Structure of the Entire Product Complex

**DOI:** 10.1371/journal.pone.0044934

**Published:** 2012-09-13

**Authors:** Michael C. Cavalier, Young-Sun Yim, Shumpei Asamizu, David Neau, Khaled H. Almabruk, Taifo Mahmud, Yong-Hwan Lee

**Affiliations:** 1 Department of Biological Sciences, Louisiana State University, Baton Rouge, Louisiana, United States of America; 2 Department of Pharmaceutical Sciences, Oregon State University, Corvallis, Oregon, United States of America; 3 NE-CAT, Cornell University, Argonne, Illinois, United States of America; Russian Academy of Sciences, Institute for Biological Instrumentation, Russian Federation

## Abstract

The pseudo-glycosyltransferase VldE catalyzes non-glycosidic C-N coupling between an unsaturated cyclitol and a saturated aminocyclitol with the conservation of the stereochemical configuration of the substrates to form validoxylamine A 7′-phosphate, the biosynthetic precursor of the antibiotic validamycin A. To study the molecular basis of its mechanism, the three-dimensional structures of VldE from *Streptomyces hygroscopicus* subsp. *limoneus* was determined in apo form, in complex with GDP, in complex with GDP and validoxylamine A 7′-phosphate, and in complex with GDP and trehalose. The structure of VldE with the catalytic site in both an “open” and “closed” conformation is also described. With these structures, the preferred binding of the guanine moiety by VldE, rather than the uracil moiety as seen in OtsA could be explained. The elucidation of the VldE structure in complex with the entirety of its products provides insight into the internal return mechanism by which catalysis occurs with a net retention of the stereochemical configuration of the donated cyclitol.

## Introduction

**Figure 1:**
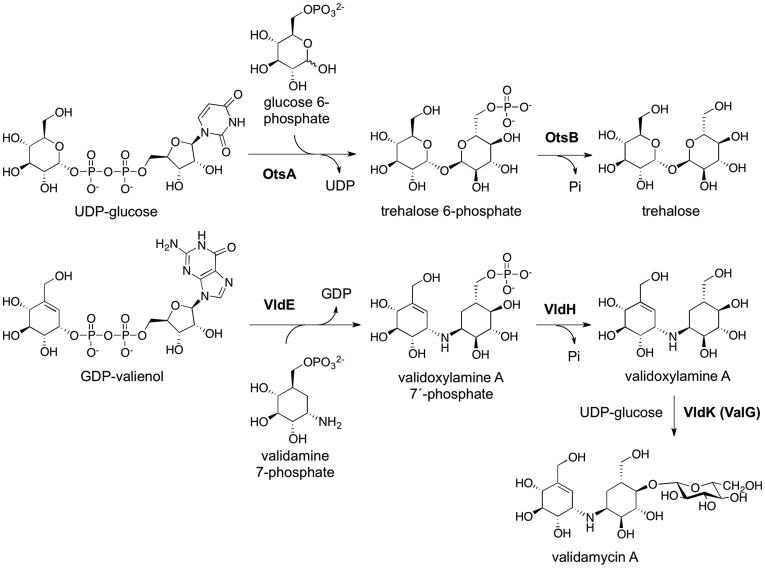
The VldE and OtsA Reactions. The product and substrates of the VldE and OtsA catalyzed reactions are shown. Note the considerable similarity between the ligands of VldE and OtsA, and the conservation of the anomeric centers. The remainders of both biosynthetic pathways are then drawn to completion. VldE catalyzes the formation of validoxylamine A 7′-phosphate via a non-glycosidic C-N bond between GDP-valienol and validamine 7-phosphate. After the validoxylamine A 7′-phosphate has been produced; VldH and VldK complete the catalytic synthesis of Validamycin A. OtsA catalyzes the formation of trehalose 6-phosphate via a glycosidic bond between UDP-glucose and glucose 6-phosphate. OtsB dephosphorylates trehalose 6-phosphate to produce trehalose.

Glycosyltransferases comprise one of the most numerous and diverse groups of enzymes in nature. They are responsible for the formation of oligo/polysaccharides, glycoproteins, glycolipids, and many other glycosylated natural products by transferring a sugar moiety from an activated donor sugar to a sugar (or non-sugar) acceptor. This abundant group of proteins consists of 92 families encoded by more than 83,400 genes [1]. However, only a fraction of those genes has actually been functionally characterized. Our comparative bioinformatics studies suggest that among those reported as glycosyltransferases are also pseudo-glycosyltransferases (such as VldE, EC 2.x.x.x), which do not recognize sugars as substrates but rather catalyze the formation of non-glycosidic C-N bonds in the biosynthesis of C_7_N-aminocyclitol-containing natural products such as acarbose and validamycin A (Figure 1) [2]–[4]. Acarbose, an α-glucosidase inhibitor, has been proven useful in the treatment of type II insulin-independent diabetes, whereas validamycin A, a natural trehalase inhibitor, is an antifungal antibiotic that has long been used to protect crops from soil borne diseases such as rice sheath blight and the dumping-off of cucumber seedlings [5]–[8].

Validamycin A is a pseudo-trisaccharide whose structure is comprised of validoxylamine A and glucose (Figure 1). The final step in validamycin A biosynthesis is the attachment of glucose to the precursory validoxylamine A by the action of the glycosyltransferase VldK (ValG) [2], [9]. Validoxylamine A is generated through the dephosphorylation of validoxylamine A 7′-phosphate by VldH while validoxylamine A 7′-phosphate (VDO) results from a condensation of GDP-valienol and validamine 7-phosphate (both are pseudo-sugars) by the pseudo-glycosyltransferase, VldE [3], [4], [10]–[14].

**Figure 2:**
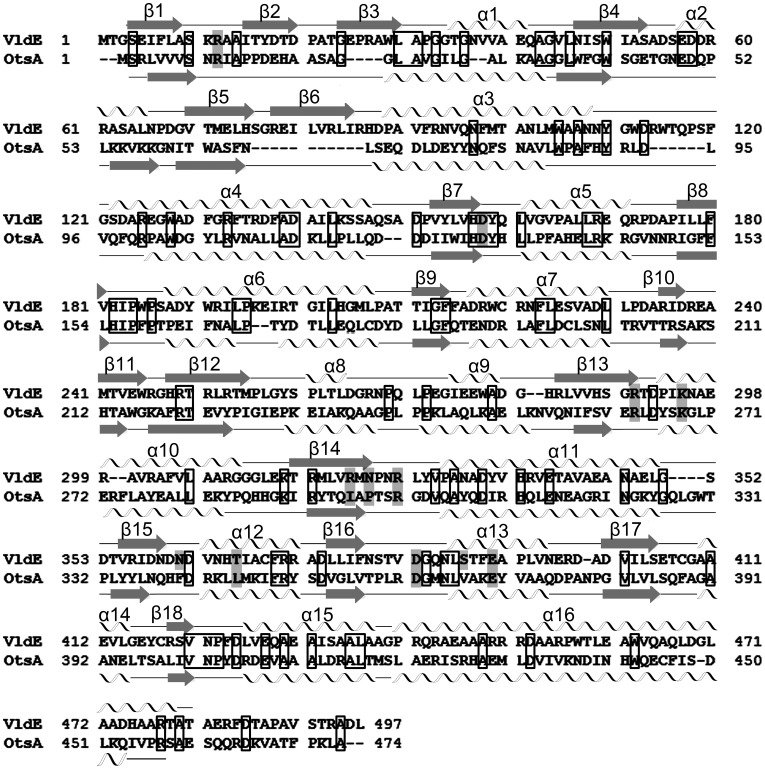
An Sequence Alignment of VldE and OtsA. Shown is the protein sequence alignment of VldE and OtsA as generated by ClustalW2 [54]. Residues whose side-chains are involved in ligand binding are indicated with shaded boxes. Conserved residues are indicated with unfilled boxes. The two-dimensional secondary structure of VldE and OtsA are also illustrated next to the corresponding sequence with α-helixes represented by spirals and β-strands represented by arrows. Numerical values for helices and strands are assigned.

The mechanism by which non-glycosidic C-N bond is formed by a pseudo-glycosyltransferase is not entirely understood. However, because of the structural similarity of validoxylamine A 7′-phosphate to trehalose 6-phosphate (Figure 1), it has been speculated that the mechanism of the pseudo-glycosyltransferase VldE is similar to that of the glycosyltransferase, trehalose 6-phosphate synthase (OtsA, EC 2.4.1.15) [4]. VldE and *Escherichia coli* OtsA only share a modest 19% sequence identity (29% homology) (Figure 2), but they are both catalogued as members of the GT20 glycosyltransferase family by the CAZy database (www.cazy.org) [15]. OtsA mediates the transfer of glucose moiety from UDP-glucose to glucose 6-phosphate to form trehalose 6-phosphate (Figure 1). Similar to VldE, the product of OtsA conserves the anomeric configuration of the donor moiety. Glycosyltransferases have been shown to both retain and invert the anomeric state of the carbon C-1 of the donor moiety. The inversion of the anomeric center by glycosyltransferases has been well explored and is known to be carried out by a simple nucleophilic substitution. However, the underlying catalytic mechanism of glycosyltransferases that retain the anomeric configuration of the donated moiety within the product is not as well understood. Catalysis by a retaining glycosyltransferases is thought to occur through either a double displacement (S_N_2 X2) or internal return mechanism (S_N_ *i*) [16]. For a double displacement nucleophilic substitution reaction to occur, a nucleophilic catalytic base must be available in close vicinity to the sugar anomeric carbon to form a covalent intermediate. OtsA apparently lacks such a residue in the catalytic site and analyses of theoretical energy profiles along with recent studies of kinetic isotope effects have substantiated this unusual enzymatic reaction mechanism within OtsA [17]–[19]. Lastly, structural studies of OtsA using a bi-substrate inhibitor as a product mimic place the atoms involved in catalysis in an orientation favorable for an internal return mechanism and show that the leaving phosphate acts as a general base to deprotonate the incoming nucleophile of the acceptor group [20]. Within the S_N_i mechanism of OtsA, a carbocation is developed upon the detachment of the nucleotide phosphate which is stabilized by resonating into an oxonium ion-like transition state [18], [20]. However, no oxonium ion formation is possible in the VldE-catalyzed non-glycosidic C-N coupling reaction. Alternatively, it is proposed that the olefinic moiety of GDP-valienol may play a critical role in facilitating the coupling reaction [4]. Coupling reactions involving an allylic moiety have been demonstrated in other biosynthetic enzymes, e.g., farnesyl diphosphate (FPP) synthases [21], [22]. However, in FPP synthases, a nucleophilic substitution reaction takes place at a carbon center with a diphosphate acting as a leaving group instead of nucleotidyl diphosphate. In addition, mechanistically FPP synthases adopt a stereospecific S_N_1 reaction with an inversion of the configuration, leaving the actual mechanism behind the unique catalytic function of the retaining VldE enzyme unclear. Hopefully, structural studies would clarify whether or not a S_N_ *i* mechanism is conserved within the retaining pseudo-glycosyltransferase, VldE.

**Figure 3:**
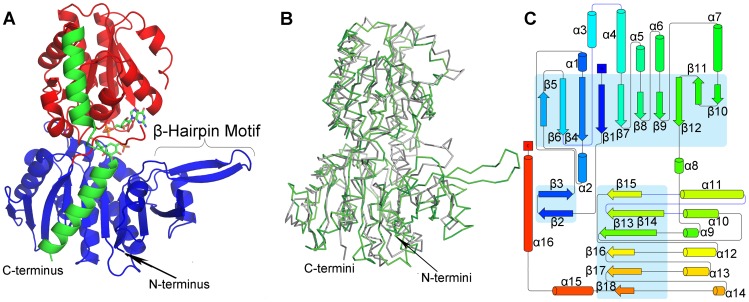
The Overall Fold of VldE. Shown is the overall fold of VldE as well as comparison to OtsA by superimposition. (**A**) The overall fold of the monomeric VldE is represented in a ribbon diagram. The monomer is rendered with the N-terminal domain in red, and the C-terminal domain in blue. The α-helical C-terminus, which stretches back across both domains, is rendered in green. VldE consists of twin Rossman-like β/α/β domains in a GT-B configuration with the catalytic site marked by VDO and GDP at the interface of the two domains. (**B**) To compare the overall fold of VldE (green) to OtsA (gray), the folding patterns, which were represented by a tracing of C^α^, were superimposed. (**C**) Shown is a topology diagram of VldE with β-strands and α-helices labeled. Blue boxes identify the core β-sheets of the N- and C-terminal Rossman domains as well as the unique β-hairpin motif.

**Figure 4:**
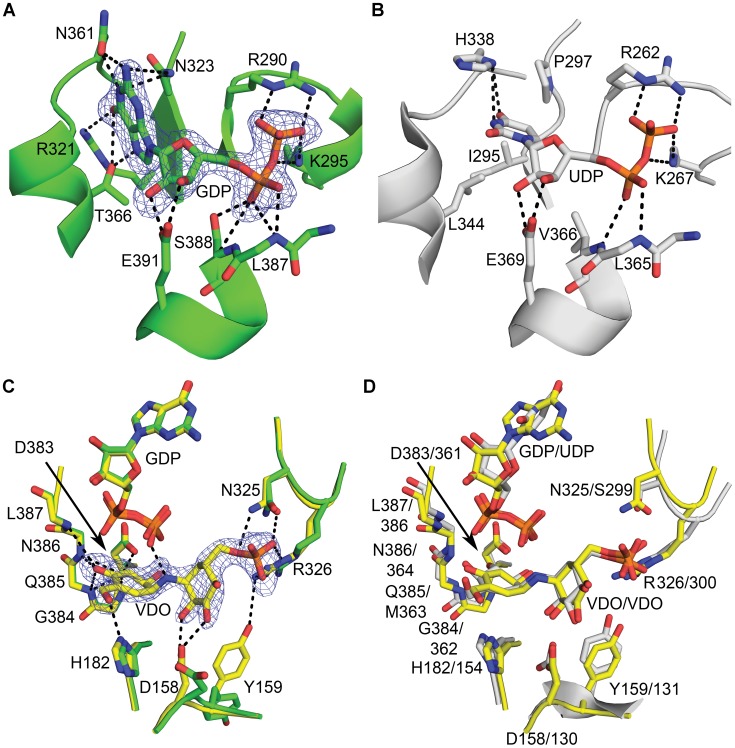
The Catalytic Site of VldE. Shown is a comparison of the VldE and OtsA catalytic sites in ribbon diagrams. Residues/molecules of interest are represented in stick models. The dotted lines mark hydrogen bonds and ionic interactions. The preferential binding of GDP by VldE is demonstrated by comparing (**A**) the protein-ligand interactions within the VldE active site (green) with (**B**) the protein-ligand interactions between OtsA and UDP within the OtsA active site (gray). The protein interactions with ribose and phosphate are conserved between VldE and OtsA. However, there are differing interactions with the nucleotide base groups. The large purine makes interactions with the residues Arg321, Asn323, Asn361, and Thr366. The ribose and phosphate moieties interact with Arg290, Lys295, Leu387, Ser388, and Glu391. Within OtsA, Leu344, Ile295, Pro297, and His338 only allow for the binding of the smaller pyrimidine. The ribose and phosphate moieties make interactions with residues Arg262, Lys267, Leu365, and Glu369. The mesh represents the |F_o_| − |F_c_| electron density omit map of the GDP binding site. The map is contoured at 3.0σ levels. (**C**) Shown is a superimposition of the VldE cyclitol binding sites in the presence (yellow) and absence (green) of validoxylamine A 7′-phosphate. The mesh represents the |F_o_| − |F_c_| electron density omit map of the VDO binding site. The map is contoured at 3.0σ levels. VDO makes interactions with residues the side-chains of residues Asp158, His182, Arg12, Asn325, Arg326, and Asp383. VDO also makes interactions with the backbones of residues 384–387. Binding of the acceptor cyclitol is recognized by conformation changes by the side-chains of residues Asp158, Tyr159, and Arg326. (**D**) Shown is a comparison by superimposition of VDO binding within the catalytic sites of VldE (yellow) and OtsA (gray). Note the strong conservation of residue and ligand positions.

Here, we report the three-dimensional structures of VldE in various liganded states using X-ray crystallographic techniques. The structure of VldE was solved by molecular replacement using the structure of OtsA as a search model. We have elucidated the structures of the unliganded VldE, in complex with guanosine 5′-diphosphate (GDP), in complex with GDP and Trehalose (TRE), and in complex with GDP and VDO. Similar to OtsA, VldE is comprised of two Rossman β/α/β domains which are oriented in a GT-B configuration (Figure 3). The active site, which is located at the interface of the Rossman domains, and the selective interactions allowing for the binding of GDP but not uridine 5′-diphosphate (UDP) are described in our study (Figure 4A–B). The crystallographic investigation of VldE while binding the complete product complex, VDO and GDP, supports the proposed conservation of a S_N_ *i* catalytic mechanism analogous to the mechanism of OtsA. Both the “open” and “closed” conformations of the catalytic site are also described within this study.

## Materials and Methods

### Purification and Crystallization

VldE from *S. hygroscopicus* subsp. *limoneus* was expressed and purified with methods similar to those previously described [4]. (His)6-tagged VldE was expressed in *E. coli* BL21 (DE3) pLysS and was purified using Ni-NTA affinity chromatography. Affinity chromatography was performed using 40 mM Hepes buffer, pH 7.5 with 300 mM NaCl, 20 mM imidazole, 5 mM β-mercaptoethanol, and 10% glycerol. Elution from the Ni-NTA resin was achieved using the same buffer but with 175 mM imidazole. Using dialysis, the affinity chromatography buffer was exchanged with VldE storage buffer consisting of 10 mM Tris-HCl, pH 7.5 with 5% glycerol, 0.1 mM dithiothreitol, and 1 mM MgCl_2_. VldE was concentrated to 10 mg/mL, and crystals were grown using sitting drop vapor diffusion.

In the case of VldE and VldE•GDP crystals, the mother liquor consisted of 100 mM Tris-HCl, pH 8.0 with 30–35% polyethylene glycol 3,350. GDP, at a concentration of 3 mM, was added to protein stocks which were intended to grow VldE•GDP crystals by co-crystallization. Crystals with a size of 0.1×0.1×0.5 mm were grown over a period of four to twelve weeks. Crystallization took place at a temperature of 293 K. Cryoprotectant solutions, consisting of artificial mother liquor enriched with 20% ethylene glycol, were added to crystal drops at a 1∶1(v/v) ratio. In the case of VldE•GDP crystals, 5 mM GDP was added to the cryoprotectant solution. Crystals were flash-cooled in liquid nitrogen after soaking in the cryoprotectant solution for 20–40 minutes.

In the case of VldE•GDP•VDO crystals, the mother liquor consisted of 100 mM Tris-HCl, pH 8.0 with 25–30% polyethylene glycol 3,350. The drop was formed through a mixture of mother liquor, protein, and seed stock in a ratio of 1.6∶2.0∶0.4 (v/v). Seed stocks were prepared from crystal grown in similar conditions using Seed Beads (Hampton Research) in a stabilization buffer of 100 mM Tris-HCl, pH 8.0 with 31% polyethylene glycol 3,350. The Crystals with a size of 0.1×0.1×0.5 mm were grown over a period of four to twelve weeks at a temperature of 293 K. Cryoprotectant solutions, consisting of artificial mother liquor enriched with 20% ethylene glycol, were added to crystal drops at a 1∶1(v/v) ratio. 5 mM GDP and 10 mM VDO were added to the cryoprotectant solutions. Crystals were flash-cooled in liquid nitrogen after soaking in the cryoprotectant solution for 20–40 minutes.

In the case of VldE•GDP•TRE crystals, the mother liquor consisted of 50 mM Tris-HCl, pH 8.0, 18–28% polyethylene glycol 3,350, 200–500 mM NaCl, and 20 mM MgCl_2_. VldE was kept at a concentration of 10 mg/mL, and crystals were grown using sitting drop vapor diffusion with a 1∶1(v/v) mixture of protein and mother liquor. Before the drops were set, 3% trehalose and 1 mM GDP were added to the protein stocks. The Crystals with a size of 0.1×0.1×0.1 mm were grown over a period of four to eight weeks at a temperature of 293 K. Cryoprotectant solutions, consisting of reservoir solution enriched with 20% ethylene glycol, 3% trehalose, and 1 mM GDP were added to crystal drops at a 1∶1(v/v) ratio. Crystals were flash-cooled in liquid nitrogen after soaking in the cryoprotectant solution for 20–40 minutes.

### Data Collection and Processing

All diffraction data except for the VldE•GDP dataset was collected at the Northeastern Collaborative Access Team (NE-CAT) beamline at the Advanced Photon Source (Argonne National Laboratory). Data was recorded at 100 K on an ADSC Q315 (315 mm×315 mm) detector and processed by NE-CAT's RAPD automated processing (https://rapd.nec.aps.anl.gov/rapd), which uses XDS [23] for integration and scaling. A 2.0 Å VldE dataset was collected at a wavelength of 0.9479 Å while oscillating the crystal 0.5 degrees for each frame. A 2.11 Å VldE•GDP•VDO dataset was collected at a wavelength of 0.9792 Å while oscillating the crystal 1.0 degrees for each frame. A 2.81 Å VldE•GDP•TRE dataset was collected at a wavelength 0.9792 Å while oscillating the crystal 1.0 degrees for each frame. The 2.15 Å VldE•GDP dataset was collected at NSLS Beamline X6A at a wavelength 1.0 Å while oscillating the crystal 1.0 degrees for each frame. X-ray data was collected on a ADSC Quanta CCD detector at 100 K and was integrated, merged, and scaled using HKL2000 [24]. Although the cell dimensions were similar, the unliganded VldE crystal belonged to the *P*2 space group, while the VldE•GDP and VldE•GDP•VDO crystals belonged to the *P*2_1_ space group. The unit cell dimensions of the VldE•GDP•TRE dataset were dissimilar and belonged to the *C*2 space group. Diffraction data statistics are summarized in Table 1.

**Table 1:** Statistics of reflection data and structure refinements.

Liganding	VldE	VldE•GDP	VldE•GDP•VDO	VldE•GDP•TRE
Space Group	*P*2	*P*2_1_	*P*2_1_	*C*2
Unit Cell Dimension (Å)	84.75, 48.43,	47.82, 121.15,	47.96, 120.71,	320.82, 122.82,
	122.66	84.33	84.24	93.86
Unit Cell β-angles(°)	91.88	91.74	91.63	91.62
Wilson Plot B Value (Å^2^)	48.74	35.96	40.37	53.40
Resolution Range (Å)	48.92–1.95	37.52–2.15	47.94–2.11	49.15–2.81
Reflections Observed	245,918	368,911	205,530	220,052
Unique Reflections	69,317	51,924	55,049	88,243
Reflections R_free_ Set	3,559	2,645	2,790	4,421
Completeness (%)	98.1 (91.8)	99.9 (100.0)	99.7 (99.0)	99.3 (98.5)
Redundancy	3.5 (3.3)	7.1 (7.1)	3.7 (3.7)	2.5 (2.5)
<I/σ>	13.0 (2.7)	25.2 (3.1)	16.5 (2.7)	12.2 (2.7)
R_sym_	0.045 (0.421)	0.078(0.522)	0.049(0.433)	0.058(0.304)
R_work_	0.190	0.197	0.191	0.211
R_free_	0.222	0.209	0.222	0.258
No. TLS Bodies	6	n/a	n/a	n/a
No. of Amino Acids	957	948	950	2,738
No. of Protein Atoms	7,474	7,378	7,361	21,796
No. of Hetero Atoms	0	58	120	214
No. of Waters	492	405	488	202
RMSD Bond Lengths (Å)	0.022	0.012	0.017	0.016
Angles (°)	1.67	1.30	1.76	1.47
Mean B Factor	41.85	40.21	39.62	62.41
Protein Atoms (Å^2^)	42.63	40.19	39.59	62.61
Hetero Atoms (Å^2^)	n/a	34.34	37.63	62.57
Water Atoms (Å^2^)	29.38	40.93	40.50	40.82
Ramachandran Outliers (%)	0.5	0.4	0.4	0.4
Ramachandran Favored (%)	97.8	97.8	97.3	95.5
Poor Rotamers (%)	1.6	1.3	1.6	1.5

R_sym_ = Σ *_h_*(Σ *_j_*|*I_hj_* − <*I_h_*>|/Σ *I_h,j_*), where _h_ = set of Miller indices, *j* = set of observations of reflection *h*, and <*I_h_*> = the mean intensity. RMSD values are deviation from ideal values. R_crys_ =  Σ*_h_*||F*_o_*_,*h*_| – |F*_c,h_*||/Σ*_h_*|F*_o,h_*|. R_free_ was calculated using 5% of the complete data set excluded from refinement. The numbers in parentheses represent values from the highest resolution shell (2.08–2.00 Å for VldE, 2.23–2.15 Å for VldE•GDP, 2.22–2.11 Å for VldE•GDP•VDO, and 2.96–2.81 Å for VldE•GDP•TRE).

**Figure 5:**
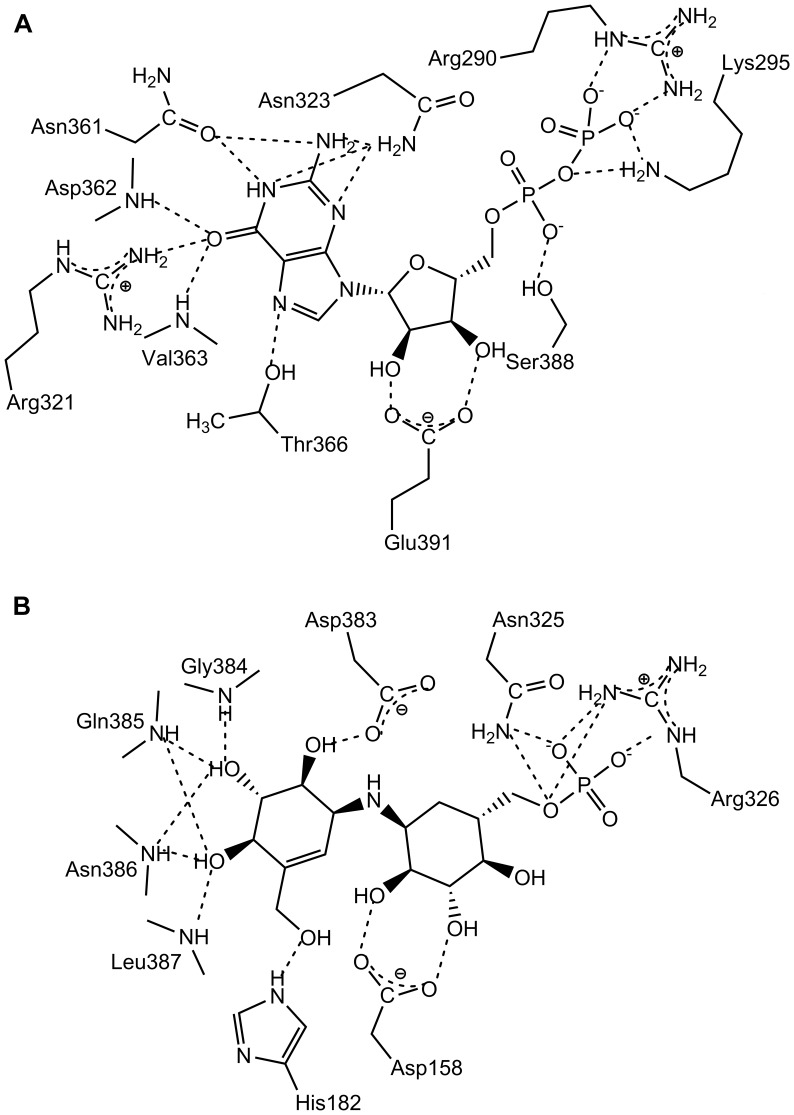
Ligand Interactions. Shown in this figure is the hydrogen bond network between VldE and (**A**) GDP and then (**B**) VDO.

### Structure Determination and Refinement

To determine the structure of VldE, molecular replacement (MR) was performed. Generation of a search model from the OtsA structure (PDB accession code **1UQU** [25]) and molecular replacement was carried out using MrBUMP [26] to orchestrate molecular replacement via the MOLREP [27] module in the CCP4 [28] program suite. The resulting MR model was used to build a partially sequenced model (∼400 residues) using the AUTOBUILD [29]–[32] function of the PHENIX [33] software suite. Further automated model building was carried out using the ARP/wARP [34] module within the CCP4 [28] software suite. The model was finished by manual building within COOT [35]. The model was refined using restrained refinement by REFMAC5 [36] with TLS parameters defined by the TLSMD server [37], [38]. Two monomers were modeled within the asymmetric unit, and non-crystallographic symmetry (NCS) restraints were used during refinement. Waters were incorporated into the models by referring to the |Fo| − |Fc| omit maps. Out of the possible 994 residues, 957 residues were modeled with 97.8% within the most favored regions of the Ramachandran plot. Although 0.5% are reported as outliers, they are all within well-defined electron densities. The final model, which lacked residues 1, 2, and 482–497, has a R_work_ of 0.190 and an R_free_ of 0.222. The structure refinement statistics are summarized in Table 1.

The VldE•GDP, VldE•GDP•VDO, and VldE•GDP•TRE structures were solved using the unliganded VldE structure as a search model within the AUTOMR [39] function of the PHENIX [33] software suite. The models were finished by manual building within COOT [35]. The models was refined the PHENIX.REFINE [29] function of the PHENIX [33] software suite. Ligands and waters were incorporated into the models by referring to the |Fo| − |Fc| omit maps. Two monomers were modeled within the VldE•GDP the asymmetric unit, six monomers were modeled within the VldE•GDP•TRE the asymmetric unit, and two monomers were modeled within the VldE•GDP•VDO the asymmetric unit. NCS restraints were used during all refinements. In these structures, 0.4% of residues are reported as Ramachandran outliers; however, they are all within well-defined electron densities, and outliers at residues Asp158 and Asn386 were common within all the monomers of all the models of VldE.

Diffraction and structure refinement statistics are summarized in Table 1. Coordinates and structure factors have been deposited in the Protein Data Bank with accession numbers: VldE (PDB ID: 3VDM), VldE•GDP (PDB ID: 4F96), VldE•GDP•VDO (PDB ID: 4F97), and VldE•GDP•TRE (PDB ID: 4F9F).

**Table 2:** Notable interactions between VldE and ligands.

		GDP		Validoxylamine A		Trehalose	
				7′-Phosphate			
Protein Atom		Guanine	Ribose 5′-	Donor	Acceptor	Donor	Acceptor
			phosphate	Cyclitol	Cyclitol-P	Sugar	Sugar
Arg321*	NH2	O6 (3.3)					
Asn323*	ND2	N1 (3.3)					
	ND2	N2 (3.4)					
Asp360	O	O6 (3.3)					
Asn361	OD1	N1 (2.6)					
	OD1	N2 (2.9)					
Asp362	N	O6 (2.9)					
	O	O6 (3.2)					
Thr366*	OG1	N7 (2.6)					
	OG1	O6 (3.6)					
Arg290	NE		O2B (2.8)				
	NH2		O1B (2.9)				
Lys295	NZ		O3A (3.1)				
	NZ		O1B (3.0)				
Leu387	N		O1A (3.4)				
	N		O2A (2.8)				
Ser388	OG		O1A (2.6)				
	N		O1A (3.1)				
Glu391	OE1		O2’ (2.5)				
	OE2		O3’ (2.6)				
His182	ND1			OAQ (2.8)		O6P (3.7)	
Asp383	OD1			OAS (2.9)		O3P (3.3)	
Gly384	N			OAS (3.0)		O3P (2.6)	
Gln385	N			OAS (2.7)		O3P (2.2)	
	N			OAR (2.8)		O4P (3.2)	
Asn386	N			OAS (3.4)		O3P (3.4)	
	N			OAR (2.7)		O4P (2.6)	
Leu387	N			OAR (3.6)		O4P (3.7)	
Asp158	OD1				OAO (3.3)		
	OD2				OAP (2.3)		
Asn325	ND2				OAX (2.4)		
	ND2				OAY (2.7)		
Arg326	NH1				OAW (3.0)		
	NE				OAY (3.6)		
Arg290	NE						O4 (3.7)

Values in parentheses are distances given in angstroms.

Donor/Acceptor is an indicator of relative position within the catalytic site.

Distances describing GDP interactions with VldE are taken from the VldE•GDP model.

* Indicates residues not conserved in OtsA that make guanine specific interactions.

## Results

### Overall Structure

As was described in a recent crystallographic study of ValL [40], a VldE homolog from *S. hygroscopicus* subsp. *jinganggensis*, VldE is a homodimer. Each monomer consists of twin Rossman β/α/β domains in the GT-B configuration [41] (Figure 3A) with the terminal helix (α16) extending back across the C-terminal Rossman-like domain to reach the N-terminal Rossman-like domain with a turn at Trp458 as it crosses the interface of the two domains. Despite a modest 19% sequence identity (29% homology) (Figure 2), the overall fold of VldE is remarkably similar to OtsA (Figure 3B), a fellow member of the GT20 family. Unlike the previous study [40] however, the core of the N-terminal domain of our apo enzyme is found with the catalytic site in an “open” conformation instead of “closed”. In this “open” conformation, the core β-sheet of the N-terminal Rossman-like domain consists of only ten strands instead of twelve. Two strands described as part of the core sheet in the previous study now are observed in a unique β-hairpin motif (β2 and β3) extending away from the catalytic site. Simultaneously, there is a dramatic shift of residues 33–47 away from the catalytic center. Unfortunately, coordinates from the structural study of ValL were not publicly available at the time of this study, so a complete comparison could not be performed. However, the closed conformation of VldE will be described in later sections of this article using our own experimental data.

**Figure 6:**
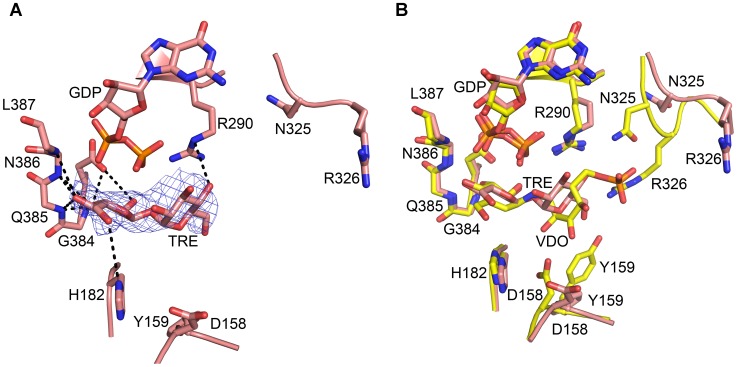
Trehalose within the Catalytic Site. Shown is a trehalose (TRE) within the VldE cyclitol binding site in ribbon diagrams. Residues/molecules of interest are represented in stick models. The dotted lines mark hydrogen bonds. (**A**) The mesh represents the |F_o_|−|F_c_| electron density omit map of trehalose within the VldE catalytic site (pink). The map is contoured at 3.0σ levels. Trehalose makes interactions with the backbone amides of residues Gly384, Gln385, Asn386, and Leu387 as well with the side-chains of Asp383, His182 and Arg290. (**B**) Shown is a superimposition of the catalytic sites of VldE·GDP·VDO model (yellow) and the VldE·GDP·TRE model (pink) in ribbon diagrams. Trehalose does not assume a binding pose comparable to VDO. This is most likely to do the absence of a phosphoryl group. Due to the absence of the phosphate moiety, Arg326 and Asn325 also swing out of the catalytic site.

**Figure 7:**
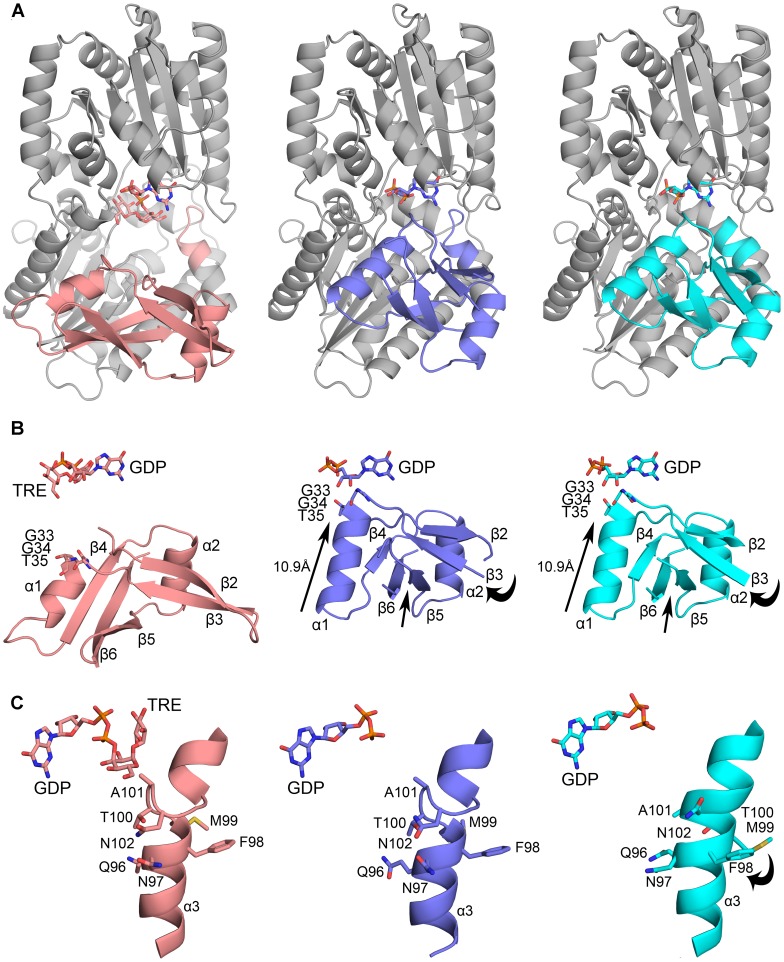
Conformational Changes. Shown are ribbon diagrams of the three conformers of VldE modeled using the VldE·GDP·TRE crystallographic data. Residues/molecules of interest are represented in stick models. Arrows indicate the direction of residue movement. Ligands bound within each conformer are shown to provide a point of reference for comparison. (**A**) The global conformation of each conformer (gray) is shown with the areas of conformational change highlighted by coloring (pink, blue, cyan). (**B**) Residues 11–50 for each conformer is shown. Note that this region of residues is capable of 10.9 Å shift towards the catalytic center and that strands of the β-hairpin motif (β2 and β3) move simultaneously with strands β6 and β5 to extend the core β-sheet from ten to twelve strands. (**C**) A view of helix α3 for each conformer is shown. Note that residues at the kink of the helix are capable of reorganizing.

**Figure 8:**
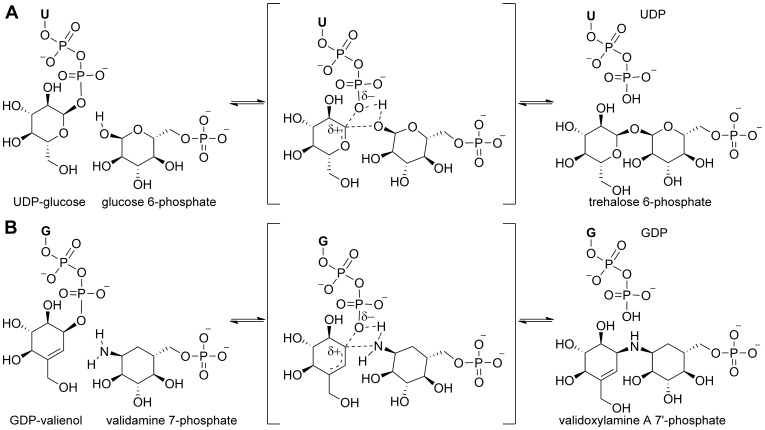
The S_N_ *i* mechanism of VldE. Shown is a figure comparing the S_N_ *i* mechanism of (**A**) OtsA to the proposed S_N_ *i* mechanism of (**B**) VldE. The olefinic moiety of GDP-valienol plays a critical role in facilitating the coupling reaction through a mechanism similar to the formation of an oxonium ion-like transition state upon the detachment of the nucleotide phosphate within OtsA. Both transition states are formed by the coordination of the allylic carbon on the donated group, the bridging nucleophile of the acceptor group and the leaving oxygen of the donor diphosphate-nucleotide.

The GDP bound structure of VldE (VldE•GDP) described here is also in an “open” conformation and the binding of GDP did not have an effect on the global conformation of the apo enzyme (RMSD C^α^ = 0.350 Å). Surprisingly, the structure of VldE in complex with the entirety of its products (VldE•GDP•VDO), GDP and VDO was also in an open conformation and a comparison to the VldE•GDP model showed that the binding of VDO also did not have an effect of the global conformation of VldE (RMSD C^α^ = 0.262 Å).

Lastly, we were also elucidated the structure of VldE in complex with both GDP and TRE (VldE•GDP•TRE). Within the VldE•GDP•TRE structure, six monomers were modeled within the asymmetric unit (ASU). The six monomers of the ASU assumed three alternative conformers. The monomers of the first conformation contained both GDP and trehalose, and this first conformer was found to also have an “open” catalytic site with similar global conformation to the VldE•GDP•VDO complex (RMSD C^α^ = 0.705 Å). The last two conformers within the VldE•GDP•TRE structure had GDP but not trehalose bound, and these two conformers contained a “closed” catalytic site as described in the previous studies [40]. These alternative conformations are significantly different when compared the “open” VldE•GDP structure (RMSD C^α^ = 2.304 Å, RMSD C^α^ = 2.395 Å) and are further discussed in later sections.

**Table 3:** DALI Server Results.

Enzyme Name	RMSD
Trehalose-Phosphate Synthase (OtsA)	2.4
*N-*Acetyl-α-D-Glucosaminyl L-Malate Synthase(*Ba*BshA)	2.9
3-Phospho-1-D-I-Inosityl-2-Acetamido-2-Deoxy-α-D-Glucopyranoside Synthase (MshA)	3.7
Trehalose Synthase (TreT)	3.1
Phosphatidylinositol Mannosyltransferase (PimA)	3.1
Glycogen Synthase (GlgA)	3.1

-Listed in order of DALI ranking.

### The Nucleotide Binding Site

Due to the conservation of key residues within their binding pockets, the binding of ligands within VldE are thought to be analogous to OtsA [19]. Here we provided experimental support for the analogous binding of GDP within VldE and UDP within OtsA. (Figure 4A) Within VldE, the ribose and phosphate moieties of GDP make interactions with the side-chains of the residues Arg290, Lys 295 and Glu391; as well as with the backbone atoms of residues Leu387 and Ser388 (Figure 5A). These interactions are conserved within OtsA (Figure 4B). The α-phosphate of GDP does make a single unique interaction with Ser388 within VldE. This interaction is not possible within OtsA as there is a valine at the same position. Nonetheless, the ribose and phosphate moieties of GDP within the VldE catalytic pocket are nearly superimposable (RMSD = 0.868) onto the ribose and phosphate moieties of UDP within the OtsA catalytic pocket. The conserved position of the β-phosphate of GDP is critically important because the bond between this phosphate and the donated cyclitol is broken during the transfer to the acceptor. As will be described later in this study, the β-phosphate of GDP maintains an interaction with the formerly bound carbon of validoxylamine A 7′-phosphate in the complete product complex (VldE•GDP•VDO). This interaction is similarly found within OtsA and similarly provides strong evidence in support of an S_N_ *i* mechanism [18].

The guanine moiety within the VldE•GDP complex makes interactions with the hydrophilic residues Arg321, Asn323, and Thr366 (Figure 5A). The preference of a GDP activated donor sugar by VldE (instead of UDP) is immediately apparent when comparing the catalytic site to that of OtsA. The preference of guanine by VldE is mediated by these three hydrophilic residues, while the preference for a uracil moiety is mediated by three hydrophobic residues at the same positions. Within OtsA, the uracil is surrounded by the hydrophobic residues Ile295, Pro297, and Leu344. This small hydrophobic binding pocket facilitates the binding of a uracil moiety. Interactions between VldE and GDP are summarized in Table 2.

### Cyclitol Binding Sites

The first crystallographic structure of VldE in complex with the entirety of its products, VDO and GDP, was elucidated (Figure 4C). The interactions between GDP and VldE are described within the previous section, but here the interactions between VDO and VldE are shown to be analogous to the interactions previously described by the OtsA•VDO complex [20]. The hydroxyls of the donated cyclitol make numerous interactions with the backbone amides of VldE at residues Gly384, Gln385, Asn386, and Leu387. The donated cyclitol also makes interactions with the side-chains of residues His182 and Asp383 (Figure 5B). The interactions by donated cyclitol are conserved within the catalytic site of OtsA (Figure 4D). Also conserved is the interaction of the β-phosphate with the formerly bound carbon of the donated cyclitol. As in OtsA, the phosphate of the leaving nucleotide is involved in the stabilization of the transition state during the concerted, front-faced nucleophilic attack of a S_N_ *i* reaction, and at a distance of 2.6 Å from the attacking nucleophile, the phosphate can act as a general base to deprotonate the amine [20].

The binding of the acceptor cyclitol within VldE is recognized by a shift in side-chain conformations within the catalytic site. Asp158 swings 62–83° towards the catalytic center in recognition of the hydroxyls of the acceptor cyclitol. Upon the binding of the phosphorylated acceptor group, the guanidine moiety of Arg326 enters into an interaction with the phosphate oxygens. This interaction allows Tyr159 to swing 110–112° to make an interaction with Arg326. The acceptor cyclitol also interacts with the side-chain of Asn325. These interactions are conserved within OtsA except at Asn325. At the same position within OtsA there is Ser299 which does not make an interaction with VDO (Figure 4D) [4].

Despite the conservation of the interactions between the catalytic site and VDO, there is no observed conformational closing of the catalytic site upon the binding of the acceptor cyclitol as was witnessed in structural studies of OtsA [19], [20], [25]. However, a closed conformation was described in the recently published structural studies of ValL [40]. In later sections, such a conformational shift will be described within our structural studies of VldE, but this shift was not triggered by the binding of the acceptor cyclitol.

We were also able to elicit the structure of VldE in complex with both GDP and trehalose (VldE•GDP•TRE). (Figure 6A) Within this model, GDP maintains the interactions previously described elsewhere within this study. The binding pose of trehalose is similar to what was described by the recent structural study of VldE [40]. The sugar moiety of trehalose within donor cyclitol binding site makes interactions with the backbone amides of residues Gly384, Gln385, Asn386, and Leu387 as well with the side-chains of Asp383 and His182. Unsurprisingly, this preserves the interactions between the enzyme and the structurally similar donor cyclitol as was described in the VldE•GDP•VDO model. However, the sugar moiety of trehalose occupying the acceptor cyclitol binding site makes only a single hydrogen bond with Arg290 in our study. A superimposition of trehalose and VDO within the VldE catalytic site shows that trehalose does not assume a binding pose comparable to VDO (Figure 6B). This is most likely due to the absence of a phosphorylated ligand. Within the VldE•GDP•VDO model, Arg326 and Asn325 interact with the phosphorylated cyclitol and most likely help to ensure the proper position of validamine 7-phosphate for catalysis. Indicatively, Arg326 and Asn325 swing out of the catalytic site within our trehalose bound structure. Interactions between VldE and VDO and TRE are summarized in Table 2.

### Observed Changes in Conformation

While solving the VldE•GDP•TRE structure, six monomers of VldE were placed within the asymmetric unit. Represented within the six monomers were three differing conformations of the N-terminal Rossman-like domain (two monomers per conformation) (Figure 7A). However, no interesting conclusions regarding possible allosteric interactions in this enzyme could be derived from these observations alone.

The first conformer had GDP and trehalose bound and is described in the previous section. This conformer is quite similar to the other models with “open” catalytic sites expect for residues 325–328 which swing out of the catalytic site upon the binding of trehalose. The second and third conformers have only GDP within the catalytic site and both of these conformers share a dramatic shift in residues 11–50 which result in the “closing” of the catalytic site (Figure 7B). Resultantly, Gly33, Gly34, and Thr35 move 10.9 Å towards the catalytic center, and within 4 Å of the ligand atoms. Additionally, helix α1, which originally consisted of residues 36–41, coils further to include residues 42–47. This shift occurs concurrently with the rotation of the β-hairpin motif (β2 and β3) unique to VldE. Additionally, the last two of ten strands (β6 and β5) comprising the core β-sheet of the N-terminal Rossman-like β/α/β domain move towards the compacting catalytic site. The movements of the unique β-hairpin motif and the two strands of the core β-sheet narrow the distance between them and allow the necessary interactions to unite them into a single twelve-stranded core β-sheet. The “closed” conformation described here is similar to what was described in the structural study of ValL. [40].

A conformational change which resulted in the closing of the catalytic site was described within the structural studies of OtsA. Residues were observed to make a >10 Å movement towards the catalytic center upon binding of the accepting sugar in OtsA [19], [25]. The conformational shift was thought to be due to the recognition of the phosphate moiety of the accepting glucose 6-phosphate by Arg9. However, with the lack of an acceptor group within the catalytic site, the conserved arginine at position 12 of VldE cannot be responsible for the closing of the catalytic site. Therefore, the closing of the VldE catalytic site is more likely to work at least partially through a more dynamic, equilibrium based mechanism.

An additional conformation change was also observed within the third conformer found within the VldE•GDP•TRE crystal (Figure 7C). Within the α3, there is a kink at residues 96–102. Within the third conformer, the kinked residues shift to form a more organized secondary structure. Again, the observed conformational change is not concerted with the binding of ligand and further supports the notion that VldE moves through equilibrium based conformations. Conformational change at this position was observed in the previous structural studies, and similarly the change seems to be focused around Gln96 [40].

## Discussion

### Mechanistic Considerations

The net retention of the substrate stereochemistry is thought to occur through either a double displacement (S_N_2 X2) or internal return mechanism (S_N_ *i*) [16]. Recent evidence substantiates claims that the conservation of the anomeric center configuration within OtsA is due to an internal return mechanism [18], [20]. If the reaction of VldE were to alternatively go through a double displacement nucleophilic substitution mechanism, a residue must be available to make a covalent intermediate. Comparatively, the hydrolysis of glycosides is known to occur through a nucleophilic substitution involving a covalent intermediate [42], [43]. The most likely candidate residue within VldE would be a nucleophilic His182. As can be seen in Figure 4D, this residue is also conserved within the catalytic site of OtsA (His154). At a 5.1 Å from the stereochemical center of the substrate, His182 is not observed to be immediately available for nucleophilic substitution in our structural studies, and as of yet there has been no significant, direct evidence to support a mechanism by which the non-glycosidic coupling reaction of VldE occurs through a covalent intermediate.

Our structural evidence demonstrates a strict conservation of nucleotide and (pseudo)sugar orientation within VldE and OtsA and suggests that the glycosyltransferase reaction of OtsA and the pseudo-glycosyltransferase reaction of VldE do occur through similar, concerted but asynchronous internal return mechanisms. Therefore, the olefinic moiety of GDP-valienol must play a critical role in facilitating the coupling reaction through a mechanism similar to the formation of an oxonium ion-like transition state upon the detachment of the nucleotide phosphate within OtsA as was previously suggested [4]. Through such a mechanism, the transition state is formed by the simultaneous binding of the allylic carbon on donated cyclitol, the bridging amine of the acceptor cyclitol-phosphate and the leaving oxygen of the donor diphosphate-nucleotide. As in OtsA [20], the leaving phosphate is in a position to deprotonate the incoming nucleophile of the acceptor cyclitol (Figure 8A–B).

Supportively, a distance matrix search using DALI [44] revealed a strong similarity between VldE and OtsA, *N-*Acetyl-α-D-Glucosaminyl L-Malate Synthase (*Ba*BshA, EC 2.4.1.x), 3-Phospho-1-D-I-Inosityl-2-Acetamido-2-Deoxy-α-D-Glucopyranoside Synthase (MshA, EC 2.4.1.250), Trehalose Synthase (TreT, EC 2.4.1.245), Phosphatidylinositol Mannosyltransferase (PimA, EC 2.4.1.57), and Glycogen Synthase (GlgA, EC 2.4.1.11) (Table 3). All are retaining glycosyltransferases that have been associated with internal return mechanisms [20], [45]–[49] (Table 3).

### Significance

The biosynthetic pathways of natural products exhibiting antibiotic-like properties have recently become of great interest. As seen in the discovery of the antibacterial erythromycin, the antihelmintic avermectins, and the antitumor indolocarbozes, the genetic manipulation of biosynthetic gene clusters along with the use of alternative biosynthetic precursors, and recombinant proteins for chemoenzymatic synthesis can result in the generation of an entire array of biologically active compounds [50]–[53]. The gene cluster responsible for the biosynthesis of validamycin A has only been recently discovered, this study and others are underway to elucidate the individual processes within this biosynthetic pathway of this C_7_N-aminocyclitol [2].

The C_7_N-aminocyclitols (e.g., acarbose, validamycin, cetoniacytone, and salbostatin) belong to a class of natural products which include the aminoglycosides (e.g., streptomycin, hygromycin, butirosin, and neomycin), and the five membered aminocyclitols or cyclopentitols (e.g., pactamycin, trehazolin, and allosamidin). The aminoglycosides are a diverse group of natural products which were amongst the first clinical antibiotics. The C_7_N-aminocyclitols are an even more diverse group of natural products with an entire range of biological activities [10]. A better understanding of the biosynthesis of C_7_N-aminocyclitols at the molecular level could allow for the generation of novel, natural product analogs which could include an array of structurally altered antibiotics. The line of inquiry provided within this article hopes to add to that understanding.

## References

[1] Hansen SF, Bettler E, Rinnan A, Engelsen SB, Breton C (2010) Exploring genomes for glycosyltransferases. Mol Biosyst 6: 1773–1781. 20556308 10.1039/c000238k

[2] Bai L, Li L, Xu H, Minagawa K, Yu Y, et al (2006) Functional analysis of the validamycin biosynthetic gene cluster and engineered production of validoxylamine A. Chem Biol. 13: 387–397. 10.1016/j.chembiol.2006.02.002 PMC1474575 16632251

[3] Singh D, Seo MJ, Kwon HJ, Rajkarnikar A, Kim KR, et al (2006) Genetic localization and heterologous expression of validamycin biosynthetic gene cluster isolated from Streptomyces hygroscopicus var. limoneus KCCM 11405 (IFO 12704). Gene 376: 13–23. 16725283 10.1016/j.gene.2005.12.035

[4] Asamizu S, Yang JT, Almabruk KH, Mahmud T (2011) Pseudoglycosyltransferase Catalyzes Nonglycosidic C-N Coupling in Validamycin A Biosynthesis. J Am Chem Soc 133: 12124–12135. 21766819 10.1021/ja203574u PMC3162038

[5] Iwasa T, Higashide E, Yamamoto H, Shibata M (1971) Studies on validamycins, new antibiotics. II. Production and biological properties of validamycins A and B. Jpn J Antibiot 24: 107–113. 10.7164/antibiotics.24.107 5549382

[6] Xia TH, Jiao RS (1986) Studies on Glutamine-Synthetase from Streptomyces-Hygroscopicus Var Jinggangensis. Sci China Ser B 29: 379–388. 2880396

[7] Iwasa T, Yamamoto H, Shibata M (1970) Studies on validamycins, new antibiotics. I. Streptomyces hygroscopicus var. limoneus nov. var., validamycin-producing organism. Jpn J Antibiot 23: 595–602. 5312765

[8] Mahmud T (2003) The C7N aminocyclitol family of natural products. Nat Prod Rep 20: 137–166. 12636088 10.1039/b205561a

[9] Xu H, Minagawa K, Bai LQ, Deng ZX, Mahmud T (2008) Catalytic analysis of the validamycin glycosyltransferase (ValG) and enzymatic production of 4 ''-epi-validamycin A. J Nat Prod. 71: 1233–1236. 10.1021/np800185k PMC2574543 18563934

[10] Mahmud T (2009) Progress in aminocyclitol biosynthesis. Curr Opin Chem Biol 13: 161–170. 19321377 10.1016/j.cbpa.2009.02.030 PMC2677119

[11] Flatt PM, Mahmud T (2007) Biosynthesis of aminocyclitol-aminoglycoside antibiotics and related compounds. Nat Prod Rep 24: 358–392. 17390001 10.1039/b603816f

[12] Mahmud T, Lee S, Floss HG (2001) The biosynthesis of acarbose and validamycin. Chem Rec 1: 300–310. 11893070 10.1002/tcr.1015

[13] Wehmeier UF, Piepersberg W (2004) Biotechnology and molecular biology of the alpha-glucosidase inhibitor acarbose. Appl Microbiol Biot 63: 613–625. 10.1007/s00253-003-1477-2 14669056

[14] Seo MJ, Im EM, Singh D, Rajkarnikar A, Kwon HJ, et al (2006) Characterization of D-glucose alpha-1-phosphate uridylyltransferase (VIdB) and glucokinase (VldC) involved in validamycin biosynthesis of Streptomyces hygroscopicus var. limoneus KCCM 11405. J Microbiol Biotechnol 16: 1311–1315.

[15] Coutinho PM, Deleury E, Davies GJ, Henrissat B (2003) An evolving hierarchical family classification for glycosyltransferases. J Mol Biol 328: 307–317. 12691742 10.1016/s0022-2836(03)00307-3

[16] Lairson LL, Henrissat B, Davies GJ, Withers SG (2008) Glycosyltransferases: Structures, functions, and mechanisms. Annu Rev Biochem 77: 521–555. 18518825 10.1146/annurev.biochem.76.061005.092322

[17] Tvaroska I (2006) Molecular modeling of retaining glycosyltransferases. Acs Sym Ser 930: 285–301.

[18] Lee SS, Hong SY, Errey JC, Izumi A, Davies GJ, et al (2011) Mechanistic evidence for a front-side, S(N)i-type reaction in a retaining glycosyltransferase. Nat Chem Biol 7: 631–638. 21822275 10.1038/nchembio.628

[19] Gibson RP, Turkenburg JP, Charnock SJ, Lloyd R, Davies GJ (2002) Insights into trehalose synthesis provided by the structure of the retaining glucosyltransferase OtsA. Chem Biol 9: 1337–1346. 12498887 10.1016/s1074-5521(02)00292-2

[20] Errey JC, Lee SS, Gibson RP, Fleites CM, Barry CS, et al (2010) Mechanistic Insight into Enzymatic Glycosyl Transfer with Retention of Configuration through Analysis of Glycomimetic Inhibitors. Angew Chem Int Edit 49: 1234–1237. 10.1002/anie.200905096 20077550

[21] Kellogg BA, Poulter CD (1997) Chain elongation in the isoprenoid biosynthetic pathway. Curr Opin Chem Biol 1: 570–578. 9667899 10.1016/s1367-5931(97)80054-3

[22] Thulasiram HV, Poulter CD (2006) Farnesyl diphosphate synthase: The art of compromise between substrate selectivity and stereoselectivity. J Am Chem Soc 128: 15819–15823. 17147392 10.1021/ja065573b PMC2516916

[23] Kabsch W (2010) Xds. Acta Crystallogr D 66: 125–132. 20124692 10.1107/S0907444909047337 PMC2815665

[24] Otwinowski Z, Minor W (1997) Processing of X-ray diffraction data collected in oscillation mode. Method Enzymol 276: 307–326. 10.1016/S0076-6879(97)76066-X 27754618

[25] Gibson RP, Tarling CA, Roberts S, Withers SG, Davies GJ (2004) The donor subsite of trehalose-6-phosphate synthase - Binary complexes with UDP-glucose and UDP-2-deoxy-2-fluoro-glucose at 2 angstrom resolution. J Biol Chem 279: 1950–1955. 14570926 10.1074/jbc.M307643200

[26] Keegan RM, Winn MD (2007) Automated search-model discovery and preparation for structure solution by molecular replacement. Acta Crystallogr D 63: 447–457. 17372348 10.1107/S0907444907002661

[27] Vagin A, Teplyakov A (1997) MOLREP: an automated program for molecular replacement. J Appl Crystallogr 30: 1022–1025.

[28] Collaborative Computational Project N (1994) The Ccp4 Suite - Programs for Protein Crystallography. Acta Crystallogr D 50: 760–763. 15299374 10.1107/S0907444994003112

[29] Afonine PV, Grosse-Kunstleve RW, Adams PD (2005) The Phenix refinement framework. CCP4 Newsletter: Contribution 8.

[30] Terwilliger TC (2004) Using prime-and-switch phasing to reduce model bias in molecular replacement. Acta Crystallogr D 60: 2144–2149. 15572767 10.1107/S0907444904019535

[31] Terwilliger TC, Grosse-Kunstleve RW, Afonine PV, Moriarty NW, Adams PD, et al (2008) Iterative-build OMIT maps: map improvement by iterative model building and refinement without model bias. Acta Crystallogr D 64: 515–524. 18453687 10.1107/S0907444908004319 PMC2424225

[32] Terwilliger TC, Grosse-Kunstleve RW, Afonine PV, Moriarty NW, Zwart PH, et al (2008) Iterative model building, structure refinement and density modification with the PHENIX AutoBuild wizard. Acta Crystallogr D 64: 61–69. 18094468 10.1107/S090744490705024X PMC2394820

[33] Adams PD, Afonine PV, Bunkoczi G, Chen VB, Davis IW, et al (2010) PHENIX: a comprehensive Python-based system for macromolecular structure solution. Acta Crystallogr D 66: 213–221. 20124702 10.1107/S0907444909052925 PMC2815670

[34] Langer G, Cohen SX, Lamzin VS, Perrakis A (2008) Automated macromolecular model building for X-ray crystallography using ARP/wARP version 7. Nat Protoc 3: 1171–1179. 18600222 10.1038/nprot.2008.91 PMC2582149

[35] Emsley P, Lohkamp B, Scott WG, Cowtan K (2010) Features and development of Coot. Acta Crystallogr D 66: 486–501. 20383002 10.1107/S0907444910007493 PMC2852313

[36] Vagin AA, Steiner RA, Lebedev AA, Potterton L, McNicholas S, et al (2004) REFMAC5 dictionary: organization of prior chemical knowledge and guidelines for its use. Acta Crystallogr D 60: 2184–2195. 15572771 10.1107/S0907444904023510

[37] Painter J, Merritt EA (2006) Optimal description of a protein structure in terms of multiple groups undergoing TLS motion. Acta Crystallogr D 62: 439–450. 16552146 10.1107/S0907444906005270

[38] Painter J, Merritt EA (2006) TLSMD web server for the generation of multi-group TLS models. J Appl Crystallogr 39: 109–111.

[39] Mccoy AJ, Grosse-Kunstleve RW, Adams PD, Winn MD, Storoni LC, et al (2007) Phaser crystallographic software. J Appl Crystallogr 40: 658–674. 19461840 10.1107/S0021889807021206 PMC2483472

[40] Zheng L, Zhou X, Zhang H, Ji X, Li L, et al (2012) Structural and Functional Analysis of Validoxylamine A 7'-phosphate Synthase ValL Involved in Validamycin A Biosynthesis. PLoS One 7: e32033. 22384130 10.1371/journal.pone.0032033 PMC3288074

[41] Vrielink A, Ruger W, Driessen HP, Freemont PS (1994) Crystal structure of the DNA modifying enzyme beta-glucosyltransferase in the presence and absence of the substrate uridine diphosphoglucose. Embo J 13: 3413–3422. 8062817 10.1002/j.1460-2075.1994.tb06646.x PMC395243

[42] Vocadlo DJ, Davies GJ, Laine R, Withers SG (2001) Catalysis by hen egg-white lysozyme proceeds via a covalent intermediate. Nature 412: 835–838. 11518970 10.1038/35090602

[43] Tews I, Perrakis A, Oppenheim A, Dauter Z, Wilson KS, et al (1996) Bacterial chitobiase structure provides insight into catalytic mechanism and the basis of Tay-Sachs disease. Nat Struct Biol 3: 638–648. 8673609 10.1038/nsb0796-638

[44] Holm L, Rosenstrom P (2010) Dali server: conservation mapping in 3D. Nucleic Acids Res 38: W545–W549. 20457744 10.1093/nar/gkq366 PMC2896194

[45] Parsonage D, Newton GL, Holder RC, Wallace BD, Paige C, et al (2010) Characterization of the N-Acetyl-alpha-D-glucosaminyl L-Malate Synthase and Deacetylase Functions for Bacillithiol Biosynthesis in Bacillus anthracis. Biochemistry 49: 8398–8414. 20799687 10.1021/bi100698n PMC2943542

[46] Vetting MW, Frantom PA, Blanchard JS (2008) Structural and enzymatic analysis of MshA from Corynebacterium glutamicum - Substrate-assisted catalysis. J Biol Chem 283: 15834–15844. 18390549 10.1074/jbc.M801017200 PMC2414306

[47] Woo EJ, Ryu SI, Song HN, Jung TY, Yeon SM, et al (2010) Structural Insights on the New Mechanism of Trehalose Synthesis by Trehalose Synthase TreT from Pyrococcus horikoshii. J Mol Biol 404: 247–259. 20888836 10.1016/j.jmb.2010.09.056

[48] Batt SM, Jabeen T, Mishra AK, Veerapen N, Krumbach K, et al (2010) Acceptor Substrate Discrimination in Phosphatidyl-myo-inositol Mannoside Synthesis: Structural and Mutational Analysis of Mannosyltransferase Corynebacterium Glutamicum PimB. J Biol Chem 285: 37741–37752. 20843801 10.1074/jbc.M110.165407 PMC2988379

[49] Horcajada C, Guinovart JJ, Fita I, Ferrer JC (2006) Crystal structure of an archaeal glycogen synthase - Insights into oligomerization and substrate binding of eukaryotic glycogen synthases. J Biol Chem 281: 2923–2931. 16319074 10.1074/jbc.M507394200

[50] Mahmud T, Flatt PM, Wu XM (2007) Biosynthesis of unusual aminocyclitol-containing natural products. J Nat Prod 70: 1384–1391. 17661520 10.1021/np070210q PMC2527543

[51] McDaniel R, Thamchaipenet A, Gustafsson C, Fu H, Betlach M, et al (1999) Multiple genetic modifications of the erythromycin polyketide synthase to produce a library of novel “unnatural” natural products (vol 96, pg 1846, 1999). P Natl Acad Sci USA 96: 5890–5890. 10.1073/pnas.96.5.1846 PMC26699 10051557

[52] Cropp TA, Wilson DJ, Reynolds KA (2000) Identification of a cyclohexylcarbonyl CoA biosynthetic gene cluster and application in the production of doramectin. Nat Biotechnol 18: 980–983. 10973220 10.1038/79479

[53] Sanchez C, Mendez C, Salas JA (2006) Engineering biosynthetic pathways to generate antitumor indolocarbazole derivatives. J Ind Microbiol Biot 33: 560–568. 10.1007/s10295-006-0092-5 16491358

[54] Larkin MA, Blackshields G, Brown NP, Chenna R, McGettigan PA, et al (2007) Clustal W and Clustal X version 2.0. Bioinformatics 23: 2947–2948. 17846036 10.1093/bioinformatics/btm404

